# Early Warning Scores Generated in Developed Healthcare Settings Are Not Sufficient at Predicting Early Mortality in Blantyre, Malawi: A Prospective Cohort Study

**DOI:** 10.1371/journal.pone.0059830

**Published:** 2013-03-29

**Authors:** India Wheeler, Charlotte Price, Alice Sitch, Peter Banda, John Kellett, Mulinda Nyirenda, Jamie Rylance

**Affiliations:** 1 College of Medical and Dental Sciences, University of Birmingham, Birmingham, United Kingdom; 2 Department of Public Health, Epidemiology and Biostatistics, University of Birmingham, Birmingham, United Kingdom; 3 College of Medicine, University of Malawi, Blantyre, Malawi; 4 Department of Medicine, Nenagh Hospital, County Tipperary, Ireland; 5 Liverpool School of Tropical Medicine, Liverpool, United Kingdom; D'or Institute of Research and Education, Brazil

## Abstract

**Aim:**

Early warning scores (EWS) are widely used in well-resourced healthcare settings to identify patients at risk of mortality. The Modified Early Warning Score (MEWS) is a well-known EWS used comprehensively in the United Kingdom. The HOTEL score (Hypotension, Oxygen saturation, Temperature, ECG abnormality, Loss of independence) was developed and tested in a European cohort; however, its validity is unknown in resource limited settings. This study compared the performance of both scores and suggested modifications to enhance accuracy.

**Methods:**

A prospective cohort study of adults (≥18 yrs) admitted to medical wards at a Malawian hospital. Primary outcome was mortality within three days. Performance of MEWS and HOTEL were assessed using ROC analysis. Logistic regression analysis identified important predictors of mortality and from this a new score was defined.

**Results:**

Three-hundred-and-two patients were included. Fifty-one (16.9%) died within three days of admission. With a cut-point ≥2, the HOTEL score had sensitivity 70.6% (95% CI: 56.2 to 82.5) and specificity 59.4% (95% CI: 53.0 to 65.5), and was superior to MEWS (cut-point ≥5); sensitivity: 58.8% (95% CI: 44.2 to 72.4), specificity: 56.2% (95% CI: 49.8 to 62.4). The new score, dubbed TOTAL (Tachypnoea, Oxygen saturation, Temperature, Alert, Loss of independence), showed slight improvement with a cut-point ≥2; sensitivity 76.5% (95% CI: 62.5 to 87.2) and specificity 67.3% (95% CI: 61.1 to 73.1).

**Conclusion:**

Using an EWS generated in developed healthcare systems in resource limited settings results in loss of sensitivity and specificity. A score based on predictors of mortality specific to the Malawian population showed enhanced accuracy but not enough to warrant clinical use. Despite an assumption of common physiological responses, disease and population differences seem to strongly determine the performance of EWS. Local validation and impact assessment of these scores should precede their adoption in resource limited settings.

## Introduction

Adverse outcomes in patients are often preceded by abnormal physiological signs [1]. Early warning scores are composite scores of these abnormalities which correlate with outcome. These tools aid physicians in identifying the critically ill in order to direct timely medical intervention [2]. The use of emergency assessment tools in developed healthcare systems is relatively well documented [2], [3], [4], as demonstrated by an agreement by the Royal College of Physicians for the universal adoption of a Nationalised Early Warning Score in the United Kingdom [5]. Conversely, their performance is not well validated in resource limited settings [6]. Addressing the low rate of adoption of triage systems in such settings [7], [8] might significantly impact on the high mortality observed in these areas of great disease burden [9], [10].

The Early Warning Score and its modified counterpart, MEWS, predict the need for hospital admission and in-hospital mortality in European cohorts [2], [11]. In South Africa, an abbreviated MEWS assessment was used to predict patient deterioration in an emergency department [12]. However, the low positive predictive value of the score in this context limited its usefulness [6]. Early Warning Scores (EWS) incorporate physiological measurements which do predict outcome [6], [12], [13] although the addition of other simple clinical parameters might further improve the sensitivity and specificity of these scores.

The HOTEL score (Hypotension, Oxygen saturation, low Temperature, ECG abnormalities, Loss of independence) [4] presents an alternative to MEWS. This score is simple and easy to calculate compared to the graded responses utilised in other early warning assessment scores [14], making its use appropriate in an emergency setting. The inclusion of ECG alongside the other routine observations was shown to be particularly predictive of mortality in an Irish cohort [4]. In sub-Saharan Africa its use as part of scoring systems is not well validated. In light of the emerging prevalence of ischaemic heart disease as a leading cause of death in sub-Saharan Africa [15], [16] this merits investigation.

The primary aim of this study was to compare the performances of the HOTEL score and MEWS in a Malawian population. The most important predictors of mortality in this population were also identified and a modified assessment score suggested.

## Methods

### Ethical Considerations

The University of Malawi College of Medicine Research and Ethics Committee (COMREC) and the Internal Ethics Review Committee at the University of Birmingham, UK, approved the study and waived the need for individual informed consent. The use of verbal consent was also approved.

### Design and Setting

A prospective observational cohort study of all adult medical patients admitted to the Queen Elizabeth Central Hospital in Blantyre, Malawi, between February 8^th^ and March 9^th^ 2012. Patients aged over 18 years were recruited from the Adult Emergency and Trauma Centre which operates as an admission unit to the largest service and referral facility in Malawi, admitting a total of 150,000 patients annually.

Practical constraints limited recruitment to weekdays 0800–1700.

### Data Collection

Data were pseudoanonymised by study number. Demographic and physiological details were collected and recorded on the study proforma by the primary investigator at enrolment. The primary investigator and other healthcare professionals who collected physiological information had received formal training to do so.

The following information was collected: time and date of admission, sex, age (years), reported HIV status (positive, negative, unknown), blood pressure (AU941 fCA,Rossmax Ltd, UK) (mmHg), percutaneous oxygen saturation (ANAPULSE 100,Ana Wiz Ltd, UK) (%), axillary temperature (°C), respiratory rate (breaths counted over one minute), mid upper arm circumference (cm), 12 lead electrocardiogram (CP100, Welch Allyn, UK), loss of independence and conscious level using the AVPU score (A for ‘alert’, V for ‘response to vocal stimuli’, P for ‘response to painful stimuli’, U for‘unconscious’). Loss of independence was defined as the inability to stand unaided (yes or no). Mid upper arm circumference was measured half-way between the acromion of the shoulder and olecranon process of the elbow using a 150 cm tape measure. ECGs were checked by two independent researchers who classified them as normal or abnormal (see Appendix S1). ECGs containing sinus tachycardia or bradycardia in isolation were recorded as normal in accordance with the original HOTEL study [4]. Discrepancies between researchers were revisited and a final status agreed upon. Patients were excluded if an ECG trace was not obtained within one hour of admission.

The date and cause of death were ascertained from medical notes.

### Statistical Analysis

The primary outcome of interest was death within 72 hours of admission (i.e. time to death ≤3 days). 72 hour mortality is an objective and clinically relevant endpoint [17], [18] and this time-frame represents a compromise between the short term mortality used in the original HOTEL study (15 mins –24 hours) and the longer term mortality of one month used in other studies.

Descriptive statistics were used to investigate patient characteristics in the two outcome groups (dead or alive) and included means (with standard deviations, SD), medians (with interquartile ranges, IQR) and frequencies with percentages. Chi-squared tests were used to compare proportions between groups. Means and medians were compared using t-tests and Mann-Whitney U tests respectively.

Individual HOTEL and MEWS scores were calculated retrospectively for each participant and the distributions of the scores in the two outcome groups were visualised using bar charts. To calculate the HOTEL score, patients received a value of +1 for each of five present abnormal measures; systolic BP<100 mmHg, oxygen saturation<90%, temperature<35°C, ECG abnormality, loss of independence (inability to stand unaided). MEWS was calculated as shown in Table 1. Sensitivities and specificities with 95% confidence intervals were calculated for each cut-point of the HOTEL and MEWS scores and receiver operating characteristic (ROC) curves plotted. The area under the ROC curves (AUROC), with 95% confidence intervals, were obtained to evaluate the accuracy of HOTEL and MEWS at predicting in-hospital mortality within three days. The positive predictive value (PPV) and negative predictive value (NPV), with 95% confidence intervals, were calculated for an optimal cut-point of each score.

**Table 1:** Calculation of the Modified Early Warning Score (MEWS).

	3	2	1	0	1	2	3
Systolic blood pressure (mmHg)	<70	70–80	81–100	101–199		≥200	
Heart rate (bpm)		<40	40–50	51–100	101–110	111–129	≥130
Respiratory rate (bpm)		<9		9–14	15–20	21–29	≥30
Temperature (°C)		<35		35–38.4		≥38.5	
AVPU score				**A**lert	Reacting to **V**oice	Reacting to **P**ain	**U**nresponsive

Each component of MEWS has an associated score ranging from 0 to 3, based on the degree of derangement of the parameter. The total score is the sum of each component: the maximum possible score is 14.

A multivariable logistic regression model was built to investigate important predictors of death in this population. Prior to fitting the main model, univariable logistic regression models were used to investigate the association between each variable and the outcome, death within three days. A model was then fitted containing all variables with p<0.25 from the univariable analyses. The model was refined using backward elimination to remove variables one at a time, based on Wald statistics, with a cut-point of p = 0.05. In order to produce an easily calculated scoring system, continuous variables were dichotomised at levels used in the HOTEL score [4], or in previous research; for example, tachypnoea (>30 breaths min^−1^, bpm) is a commonly used predictor of mortality [2], [12]. Conscious level was condensed to ‘alert or abnormal’; abnormal being ‘V’, ‘P’ or ‘U’ from the AVPU score as previously suggested [12]. Apparent model performance was assessed using the Hosmer-Lemeshow goodness of fit test [19] and the *c*-statistic, which is equivalent to the AUROC for binary outcomes. An optimism adjusted *c*-statistic was obtained using bootstrap validation of the model with 1000 bootstrap samples.

Regression coefficients from the final model were used to generate a new score coined the TOTAL score. A ROC curve was constructed for TOTAL and the area under the curve, with 95% confidence interval, was obtained to evaluate its accuracy at predicting in-hospital mortality within three days. Its performance was compared to both HOTEL and MEWS.

Statistical analyses were carried out using IBM SPSS Statistics version 19 and R version 2.10.1.

## Results

During the study, 361 patient observations were taken. Nineteen patient records (5.3%) were excluded due to non-admission to the medical wards and 11 patient records (3.0%) due to missing data on one or more variables. A further 29 records (8.0%) were excluded due to patients being discharged less than 3 days after admission, thus excluding those who may have died at home within this period. There were 302 patient records (83.7%) in the final analyses. The flow of patients through the study is shown in Figure S1.

The mean age was 39.5 years (SD 15.9 years). A total of 155 (51.3%) patients were male and 180 (59.6%) patients were known to be HIV positive. Fifty-one patients (16.9%) died ≤3 days after admission; the median time from admission to death was 2 days (IQR 1 to 5 days). The most common cause of death was due to a respiratory complaint (n = 19, 37.3%).

### Physiological Parameters on Admission

Table 2 presents the demographic and physiological characteristics on admission by outcome. Patients who died had significantly lower oxygen saturation (p<0.001), lower temperature (mean difference: −0.51; 95% CI: −0.91 to −0.11; p = 0.012) and higher respiratory rate (mean difference: 6.02; 95% CI: 2.60 to 9.45; p = 0.001). Patients who died more commonly had reduced conscious level (p<0.001), were less frequently able to stand unaided (p<0.001) and more likely to have an ECG abnormality (p = 0.019). There was no significant difference in systolic blood pressure (mean difference: 6.61; 95% CI: −2.12 to 15.34; p = 0.137).

**Table 2:** Patient characteristics on admission by outcome group; n = 302.

Characteristic			All (n = 302)	Dead (n = 51)	Alive (n = 251)	p-value
Age (years); mean (SD)			39.5 (15.9)	39.4 (14.2)	39.6 (16.3)	0.930
Sex*; male (n, %)			155 (51.3)	29 (56.9)	126 (50.2)	0.753
HIV status* (n, %):	Positive		180 (59.6)	28 (54.9)	152 (60.6)	0.566
	Negative in last year		77 (25.5)	13 (25.5)	64 (25.5)	
	Unknown		45 (14.9)	10 (19.6)	35 (13.9)	
Systolic blood pressure (mmHg);mean (SD)			114.0 (28.9)	114.0 (28.9)	112.8 (26.9)	0.137
Oxygen saturation#; median (IQR)			96 (92 to 98)	92 (80 to 97)	97 (93 to 98)	<0.001
Axillary temperature (°C); mean (SD)			37.0 (1.3)	36.5 (1.5)	37.1 (1.3)	0.012
Respiratory rate (breaths per min);mean (SD)			29.2 (11.5)	34.2 (13.5)	28.2 (10.9)	0.001
Pulse rate (beats per min);mean (SD)			105.0 (25.6)	99.5 (27.9)	106.2 (25.0)	0.087
Mid upper arm circumference (cm); mean (SD)			22.8 (3.8)	22.1 (4.0)	23.0 (3.7)	0.115
ECG abnormal*; n(%)			89 (29.5)	22 (43.1)	67 (26.7)	0.019
Loss of independence*; n(%)			187 (61.9)	44 (86.3)	143 (57.0)	<0.001
Conscious level*; n(%):		Alert	253 (83.8)	33 (64.7)	220 (87.6)	<0.001
		Voice	33 (10.9)	10 (19.6)	23 (9.2)	
		Pain	9 (3.0)	5 (9.8)	4 (1.6)	
		Unresponsive	7 (2.4)	3 (5.9)	4 (1.6)	

SD, standard deviation; IQR, interquartile range.

t-tests were used to test differences between the alive and dead groups unless otherwise indicated;

* Chi-squared test;

# Mann-Whitney U test.

### Comparison of HOTEL and MEWS in Predicting Mortality within a Malawian Population

Figure S2 illustrates the frequency distributions of the HOTEL and MEWS scores in the two outcome groups (dead and alive). Most frequent HOTEL score was 1 for those who survived (n = 101, 40.2%), and 2 for patients who died (n = 18, 35.3%). The only patient who had a HOTEL score of 5 died within 24 hours. The most frequent MEWS score was 4 for those who survived (n = 46, 18.3%) and 6 for patients who died (n = 10, 19.6%).

Table 3 shows the sensitivities and specificities with 95% confidence intervals for HOTEL and MEWS at select cut-points. Optimal discrimination using HOTEL is found at a score ≥2 which gives sensitivity 70.6% (95% CI: 56.2 to 82.5) and specificity 59.4% (95% CI: 53.0 to 65.5). Using the optimal cut-point of score ≥5, MEWS performs less well with sensitivity 58.8% (95% CI: 44.2 to 72.4) and specificity 56.2% (95% CI: 49.8 to 62.4). At these thresholds, MEWS has positive predictive value (PPV) 21.4% (95% CI: 14.9 to 29.2) and NPV 87.0% (95% CI: 80.9 to 91.8), while HOTEL is a better discriminator; PPV 26.1% (95% CI: 19.0 to 34.2) and NPV 90.9% (95% CI: 85.4 to 94.8). The performance accuracy is further illustrated by the ROC curves (Figure S3). AUROCs were: MEWS = 0.59 (95% CI: 0.51 to 0.68) and HOTEL = 0.70 (95% CI: 0.62 to 0.78). While it is evident that HOTEL is more accurate than MEWS at predicting in-patient mortality in this population, clinical usefulness is limited at these levels.

**Table 3:** Sensitivities and specificities (in percentages) with 95% confidence intervals for MEWS, HOTEL and TOTAL at select cut-points (n = 302).

Score (range)	Cut-point	Frequency	%	Sensitivity (95% CI)		Specificity (95% CI)	
**MEWS (0 to 14)**	≥1	295	97.7	98.0	(89.6, 100.0)	2.4	(0.9, 5.1)
	≥2	268	88.7	92.2	(81.1, 97.8)	12.0	(8.2, 16.6)
	≥3	234	77.5	86.3	(73.7, 94.3)	24.3	(19.2, 30.1)
	≥4	192	63.6	70.6	(56.2, 82.5)	37.8	(31.8, 44.2)
	≥5	140	46.4	58.8	(44.2, 72.4)	56.2	(49.8, 62.4)
	≥6	93	30.8	45.1	(31.1, 59.7)	72.1	(66.1, 77.6)
	≥7	56	18.5	25.5	(14.3, 39.6)	82.9	(77.6, 87.3)
	≥8	30	9.9	13.7	(5.7, 26.3)	90.8	(86.6, 94.1)
**HOTEL (0 to 5)**	≥1	251	83.1	94.1	(83.8, 98.8)	19.1	(14.4, 24.5)
	≥2	138	45.7	70.6	(56.2, 82.5)	59.4	(53.0, 65.5)
	≥3	44	14.6	35.3	(22.4, 49.9)	89.6	(85.2, 93.1)
	≥4	8	2.6	11.8	(4.4, 23.9)	99.2	(97.2, 99.9)
	≥5	1	0.3	2.0	(0.5, 10.4)	100.0	(98.5, 100.0)
**TOTAL (0 to 6)**	≥1	236	78.1	98.0	(89.6, 100.0)	25.9	(20.6, 31.8)
	≥2	121	40.1	76.5	(62.5, 87.2)	67.3	(61.1, 73.1)
	≥3	49	16.2	43.1	(29.3, 57.8)	89.2	(84.7, 92.8)
	≥4	11	3.6	19.6	(9.8, 33.1)	99.6	(97.8, 100.0)
	≥5	1	0.3	2.0	(0.5, 10.4)	100.0	(98.5, 100.0)
	≥6	1	0.3	2.0	(0.5, 10.4)	100.0	(98.5, 100.0)

### Predicting Mortality within Three Days of Admission

In the univariable analyses (Table 4); low temperature (<35°C) was associated with a 9.15-fold increase in odds of inpatient mortality (95%CI: 2.86 to 29.30; p<0.001), thus making it one of the strongest predictors. Reduced conscious level was also very predictive with an unadjusted odds ratio (OR) of 3.87 (95% CI: 1.95 to 7.69; p<0.001) when compared to fully alert. Also strongly associated with in-patient mortality were hypoxia (oxygen saturation <90%) (OR 3.92; 95% CI: 2.00 to 7.71; p<0.001), tachypnoea (respiratory rate >30 bpm) (OR 2.26; 95%CI: 1.23 to 4.17; p = 0.009) and the inability to stand unaided (OR 4.75; 95% CI: 2.06 to 10.95; p<0.001). ECG abnormality showed a weaker, but significant, association with mortality (OR 2.08; 95%CI: 1.12 to 3.88; p = 0.020), but known HIV positive status did not (OR 0.91; 95%CI: 0.44 to 1.86; p = 0.790).

**Table 4:** Unadjusted odds ratios for demographic and physiological variables; n = 302.

				Univariable Analysis		
Variable	HOTEL	MEWS	TOTAL	OR	95% CI	p-value
Age (years)				1.00	(0.98, 1.02)	0.930
Sex(reference: female)				1.31	(0.71, 2.40)	0.390
HIV status (reference: negative):						
Positive				0.91	(0.44,1.86)	0.790
Unknown				1.41	(0.56, 3.54)	0.470
Mid Upper Arm Circumference (cm)				0.93	(0.86, 1.02)	0.120
Pulse rate (>120 beats per min)		✓		1.22	(0.62, 2.43)	0.570
Systolic Blood Pressure (<100 mmHg)	✓	✓		0.89	(0.47, 1.70)	0.730
ECG abnormality	✓			2.08	(1.12, 3.88)	0.020
Respiratory rate (>30 breaths per min)		✓	✓	2.26	(1.23, 4.17)	0.009
Oxygen saturation (<90%)	✓		✓	3.92	(2.00, 7.71)	<0.001
Temperature (<35°C)	✓	✓	✓	9.15	(2.86,9.30)	<0.001
Conscious level (reference: alert):		✓	✓			
Abnormal				3.87	(1.95, 7.69)	<0.001
Loss of independence	✓		✓	4.75	(2.06,10.95)	<0.001

The final multivariable logistic model contained five variables (see Table 5): respiratory rate>30 bpm (OR 2.14; 95% CI: 1.04 to 4.42; p = 0.039), oxygen saturation<90% (OR 3.22; 95% CI: 1.47 to 7.05; p = 0.004), temperature<35°C (OR 10.33; 95% CI: 2.90 to 36.86; p<0.001), reduced conscious level (OR 3.25 95% CI: 1.47 to 7.16; p = 0.003) and loss of independence (OR 3.24; 95% CI: 1.30 to 8.07; p = 0.012). The Hosmer-Lemeshow goodness of fit test demonstrated good calibration (p = 0.860). The model also had good discrimination with an apparent *c*-statistic of 0.794 and an optimism adjusted *c*-statistic of 0.778 after using bootstrap validation. This suggests that it performs well at distinguishing patients who died from those who did not.

**Table 5:** Adjusted odds ratios for demographic and physiological variables used in the TOTAL score; n = 302.

Variable	Coefficient	(95% CI)	OR	(95% CI)	p-value	Score*
Respiratory rate (>30 breaths per min)	0.76	(0.04, 1.49)	2.14	(1.04, 4.42)	0.039	1
Oxygen saturation (<90%)	1.17	(0.38, 1.95)	3.22	(1.47, 7.05)	0.004	1
Temperature (<35°C)	2.34	(1.06, 3.61)	10.33	(2.90, 6.86)	<0.001	2
Conscious level (reference: alert)						
Abnormal	1.18	(0.39, 1.97)	3.25	(1.47, 7.16)	0.003	1
Loss of independence	1.18	(0.26, 2.09)	3.24	(1.30, 8.07)	0.012	1
Intercept	−3.48	(−4.39, −2.58)	−	−	<0.001	−

* The score for each component of TOTAL was obtained by rounding each regression coefficient to the nearest integer value. The TOTAL score ranges from 0 to 6.

The five parameters from the final regression model were used to describe a new score, coined TOTAL (Tachypnoea, Oxygen saturation, Temperature, Alert and Loss of independence). The scores for each parameter were obtained by rounding the associated regression coefficient to the nearest integer; each abnormal reading scores +1 except temperature which is allocated +2 points due to its larger regression coefficient. The TOTAL score ranges from 0 to 6 and the distribution of the scores in the two outcome groups (dead and alive) is shown in Figure S4.

### Performance Accuracy of TOTAL

The performances of MEWS, HOTEL and TOTAL are compared in Table 2. The optimum cut-point for TOTAL (≥2), gives sensitivity and specificity 76.5% (95% CI: 62.5 to 87.2) and 67.3% (95% CI: 61.1 to 73.1) respectively, with corresponding PPV 32.2% (95% CI: 24.0 to 41.3) and NPV 93.4% (95%CI: 88.7 to 96.5). The TOTAL score outperforms both HOTEL and MEWS in terms of sensitivity and specificity, as illustrated by the ROC curves (Figure S3). The AUROC for TOTAL was 0.78 (95% CI: 0.71 to 0.85).

## Discussion

Previous research has shown the benefits of comprehensive triage assessment [2], [4], [6], [9]–[12]. This study compared two early warning assessment scores, HOTEL and MEWS, and found some evidence that HOTEL is superior to MEWS in a Malawian population, but also that further modifications may lead to modest improvements in predictive accuracy.

The average age of participants in this study was considerably younger than those reported in studies using HOTEL and MEWS in developed healthcare environments, where almost no patients under the age of 50 died [1], [2], [4]. This situation was also observed in research conducted in study settings similar to Malawi and is likely to be explained by the lower life expectancies in these countries [10].

The factors most associated with in-patient mortality within three days were tachypnoea, hypoxia, low temperature, deterioration in conscious level and inability to stand unaided. The inclusion of respiratory rate is perhaps to be expected, as its lack of value in previous research [4] was suggested to be due to inaccurate recordings by the research team. Surprisingly, HIV status was not identified as an important predictor of death. This is perhaps because although the frequency of ailments such as pneumonia is higher in seropositive individuals, this does not necessarily translate into an increased case fatality rate if appropriate treatment is available [20].

The importance of conscious level and loss of independence as predictors of mortality [6], [12] is particularly useful in this context as both observations can be recorded without equipment and can be ascertained quickly and objectively in the emergency department by individuals with minimal training [6].

In contrast, some previously identified important predictors of death were found to be less important in this population. Hypotension, for example, has been shown to be an independent predictor of in-hospital mortality [4], [6], [12], [21] and an important trigger for Intensive Care Unit admission [22], but our study found no evidence of this. This could be explained by the small scale of the study, or because hypotension in this context may be related to treatable disease processes, such as malaria or bacterial infection [6]. Secondly, hypotension is a relatively late sign of physiological derangement [23] and may be missed without serial measurements. However, a recent study in Uganda found an association between a reduced mean arterial pressure and mortality [24], and this relationship could also apply in our population.

Similarly, a normal ECG tracing has previously been documented as a strong predictor of survival in a European cohort [4]. In this study, abnormal ECG was associated with increased risk of mortality in the univariable analysis, but its predictive ability was lost after adjusting for the confounding effects of other variables. Further analysis of the effect of individual ECG abnormalities on patient outcome may be useful, although a differing prevalence of ischaemic heart disease may explain this discrepancy.

These findings demonstrate that the usefulness of early warning scores depends on the situation in which they are used.

It is perhaps unsurprising that the HOTEL score, which was shown to accurately identify at risk patients in an Irish cohort [4] (AUROC: 0.85, 95% CI: 0.75 to 0.96), did not achieve the same predictive accuracy in this setting (AUROC: 0.70. 95% CI: 0.62 to 0.78) and the low PPV (26.1%) precludes its adoption into clinical practice. Interestingly, MEWS, which is relied on extensively in well-resourced healthcare settings [2], [13], [14], had a weaker performance in the Malawian population compared to HOTEL. This may in part be due to the inclusion of oxygen saturation in the HOTEL score which supports calls for its increased clinical use due to its ease of application and low cost.

This study presents evidence for the use of population-specific predictors of mortality in adults. In comparison to HOTEL and MEWS, TOTAL had a higher specificity allowing resources to be more effectively managed. This is of critical importance to the sustainable adoption of risk scores in low income countries [25]. The balance between identifying high risk individuals yet not overburdening health services is essential in this setting and ultimately determines the suitability of such a tool. Some assessment tools such as the Integrated Management of Childhood Illness [26], which is utilised in the community, work on a ‘rule out’ basis, identifying those who do not have a defined illness and treating all others [27]. In contrast, the MEWS, HOTEL and TOTAL scores, when applied to medical patients are ‘rule in’ systems relying on high positive predictive value; they identify the patients most in need of immediate intervention. In our cohort, TOTAL had a PPV of 32.2%, which was higher than HOTEL and MEWS. Although a higher PPV is desirable, as it stands TOTAL would identify more than half of the total patient population at high risk and, on average, for every three patients identified as high risk, one death would be expected.

Our findings suggest that risk scores work best in the populations from which they were derived. We have shown that adopting an early warning score generated in a developed healthcare setting into a contrasting environment is likely to impact negatively on performance, despite the assumption that physiological responses to disease are common to all patients. This apparent discrepancy between predictors of mortality in different populations could be explained by a number of theories. For example, specific disease processes may impact more on outcome than the physiological effect they exert and physiological responses to disease may be highly dependent on age as our patients were considerably younger than the population studied in the previous HOTEL cohort.

In our hospital, we feel that there is considerable room for improvement in the service despite limited resources. This might be through a better focus of the available personnel and improved efficiency in identifying ill patients to reduce time to treatment. A well-performing scoring system could help with this. Therefore, we suggest that local data collection, looking at individual physiological predictors, should occur in the locale intended for EWS implementation before such systems are adopted. This should occur alongside impact assessment studies to investigate whether the use of a prognostic score results in an improvement in doctors’ decision making and ameliorated patient outcome [28]. This will encourage an evidence based practice approach to emergency medicine in under-resourced settings, and will enhance our understanding of the physiology of disease in different populations.

### Limitations

As observations were only taken within office hours, patients admitted during the night and weekends were not represented, yet previous research has demonstrated that admissions during these times carry a greater risk of mortality [29], [30]. Patients were also excluded from analysis if they had been discharged less than three days after admission, resulting in roughly 10% of patient records being discarded. However, since this group of patients had unknown outcomes, removing them prior to analysis ensured accuracy in the statistical calculations and models.

This study was fairly small and since the TOTAL score was developed on this dataset, its reported accuracy is likely to be optimistic. We compensated for this by using bootstrap validation to provide an optimism adjusted estimate of performance, but external validation on larger unseen datasets is required in order to evaluate its true predictive accuracy in the Malawian population.

Finally, while the TOTAL score demonstrates a modest improvement in high risk patient identification, the incremental benefit over MEWS and HOTEL is not enough for robust triage or resource allocation in clinical use. Instead, further clinical parameters, with possible inclusion of laboratory tests results such as rapid diagnostic tests for malaria, offer the potential to considerably improve usefulness of EWS, and should be investigated in this context.

The need for cost effective comprehensive adult emergency assessment systems to be implemented in low income settings remains a priority. We have demonstrated that EWS generated in developed healthcare systems do not have the same predictive accuracy in differing populations. Modified scores, such as the TOTAL score, tailored to the unique predictors of that population, show an improved performance and could also have the potential for adoption in similar patient populations. However, we appreciate that, currently, TOTAL is a not a clinically useful tool and further investigation of different clinical observations and studies on larger datasets are necessary in order to create an accurate assessment tool with real clinical use.

## Supporting Information

Figure S1 **Flow of patients through the study; n = 302.**
(TIF)

Figure S2 **Distribution of the HOTEL and MEWS scores across the two outcome groups (alive and dead); n = 302.**
(TIF)

Figure S3 **Receiver operator characteristic (ROC) Curves for the HOTEL, MEWS and TOTAL scores (n = 302).** The solid line shows the line of no discrimination where the test is no better than chance.
(TIF)

Figure S4 **Distribution of the TOTAL scores across the two outcome groups (dead and alive); n = 302.**
(TIF)

Appendix S1 **Classification table of ECG abnormalities.**
(DOCX)
