# Correction: Early Warning Scores Generated in Developed Healthcare Settings Are Not Sufficient at Predicting Early Mortality in Blantyre, Malawi: A Prospective Cohort Study

**DOI:** 10.1371/journal.pone.0091623

**Published:** 2014-02-28

**Authors:** The PLOS ONE Staff

In the Author Contributions Statement, the sixth author (MN) should be noted as one of the authors who "Wrote the paper."

## References

[1] Wheeler I, Price C, Sitch A, Banda P, Kellett J, et al (2013) Early Warning Scores Generated in Developed Healthcare Settings Are Not Sufficient at Predicting Early Mortality in Blantyre, Malawi: A Prospective Cohort Study. PLoS ONE 8(3): e59830 doi:10.1371/journal.pone.0059830 23555796 10.1371/journal.pone.0059830 PMC3612104

