# Supplementary figures and images for: Early Warning Scores Generated in Developed Healthcare Settings Are Not Sufficient at Predicting Early Mortality in Blantyre, Malawi: A Prospective Cohort Study
Source: PLoS One. 2013 Mar 29;8(3):e59830. doi: 10.1371/journal.pone.0059830 (PMC3612104; doi:10.1371/journal.pone.0059830)

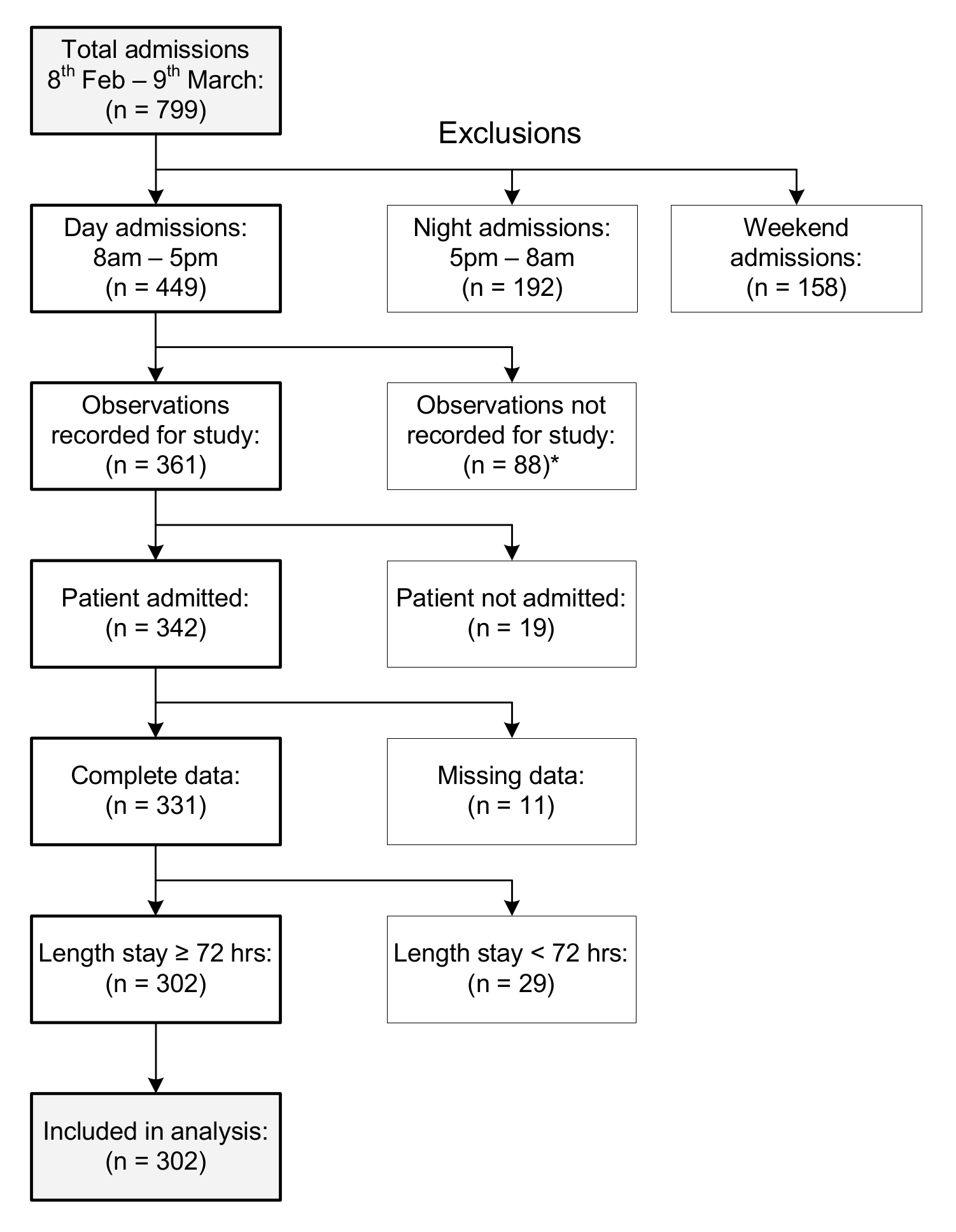

Supplement: Figure S1 — Flow of patients through the study; n = 302. (TIF) [file pone.0059830.s001.tif]

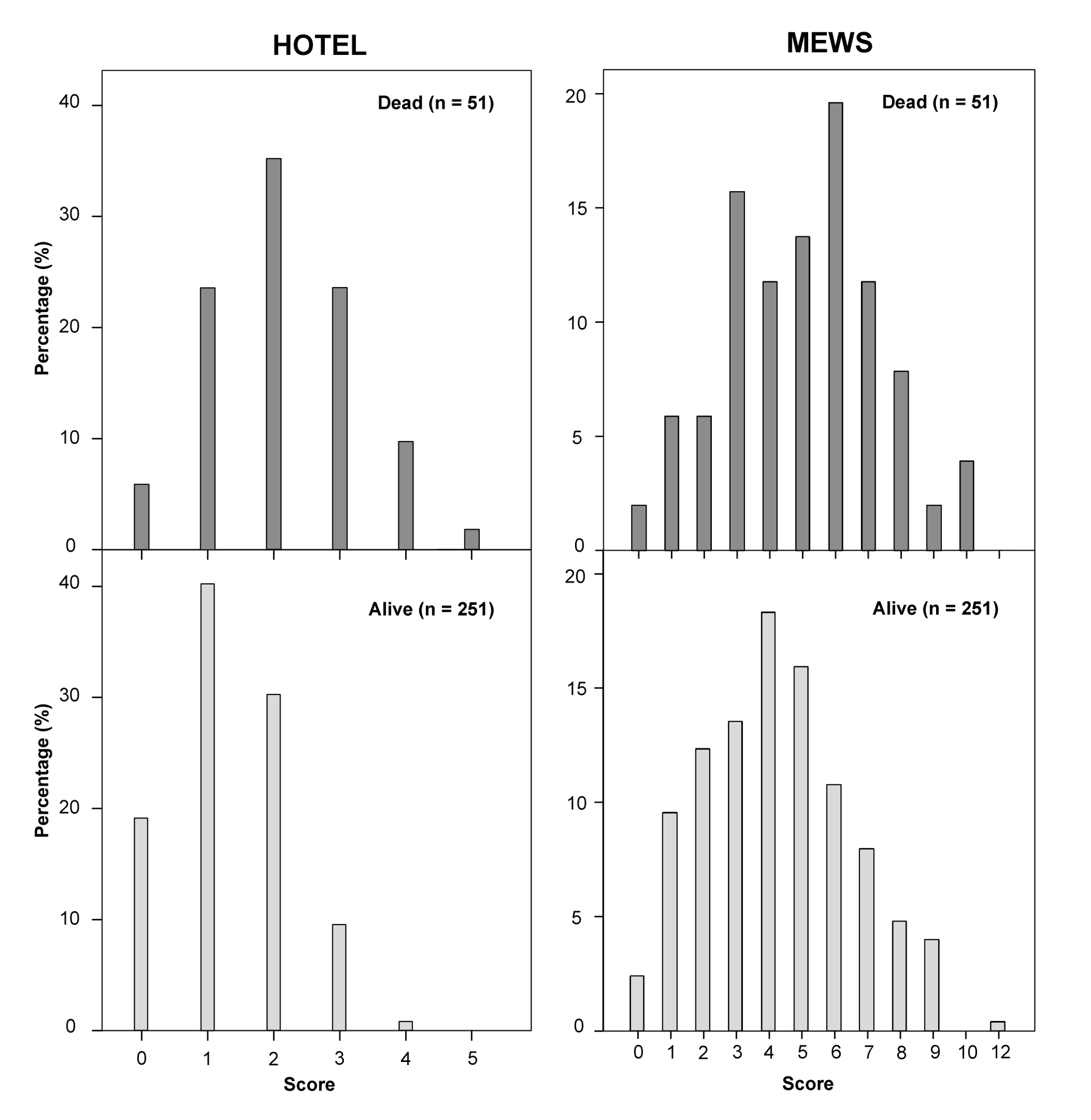

Supplement: Figure S2 — Distribution of the HOTEL and MEWS scores across the two outcome groups (alive and dead); n = 302. (TIF) [file pone.0059830.s002.tif]

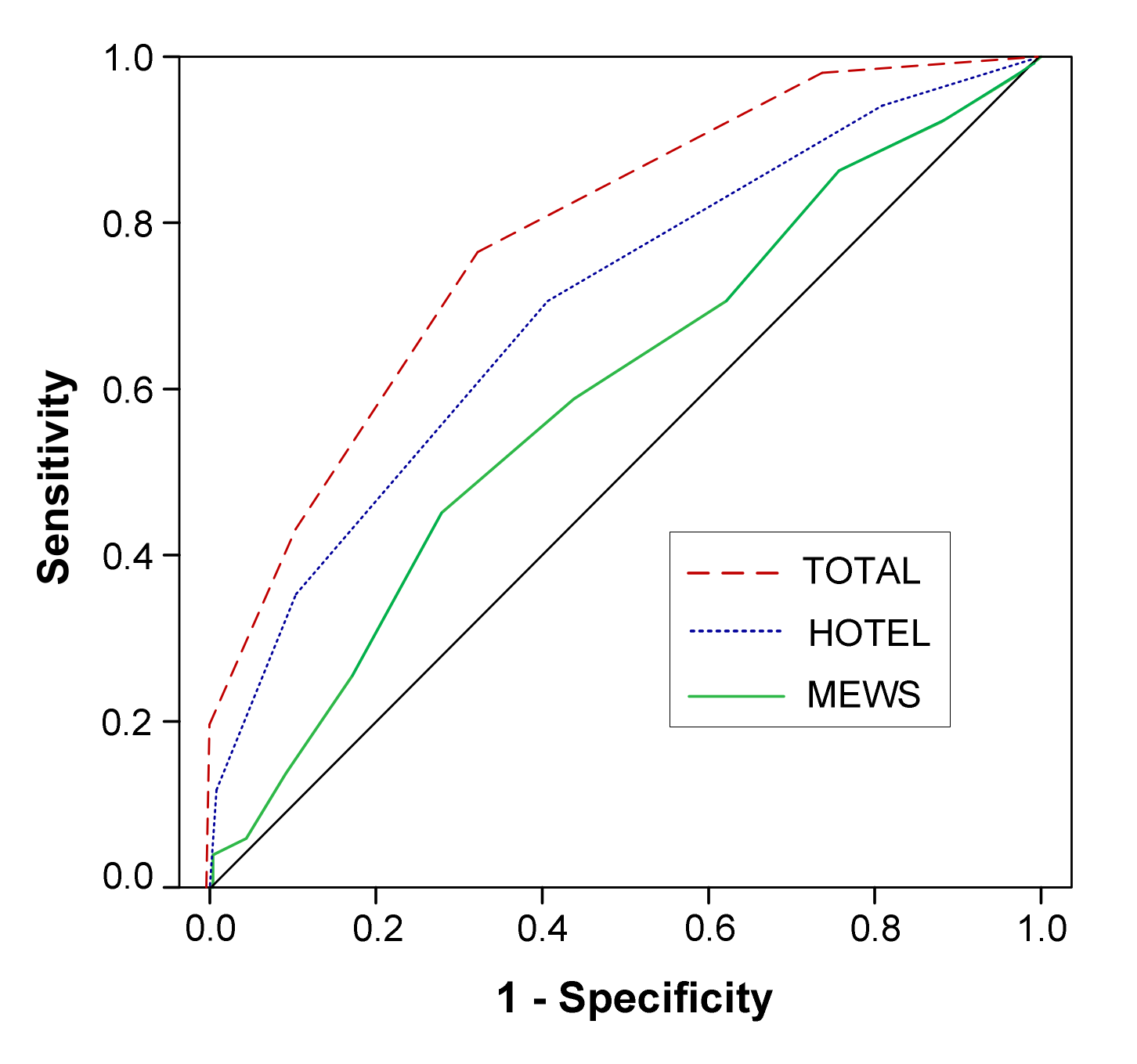

Supplement: Figure S3 — Receiver operator characteristic (ROC) Curves for the HOTEL, MEWS and TOTAL scores (n = 302). The solid line shows the line of no discrimination where the test is no better than chance. (TIF) [file pone.0059830.s003.tif]

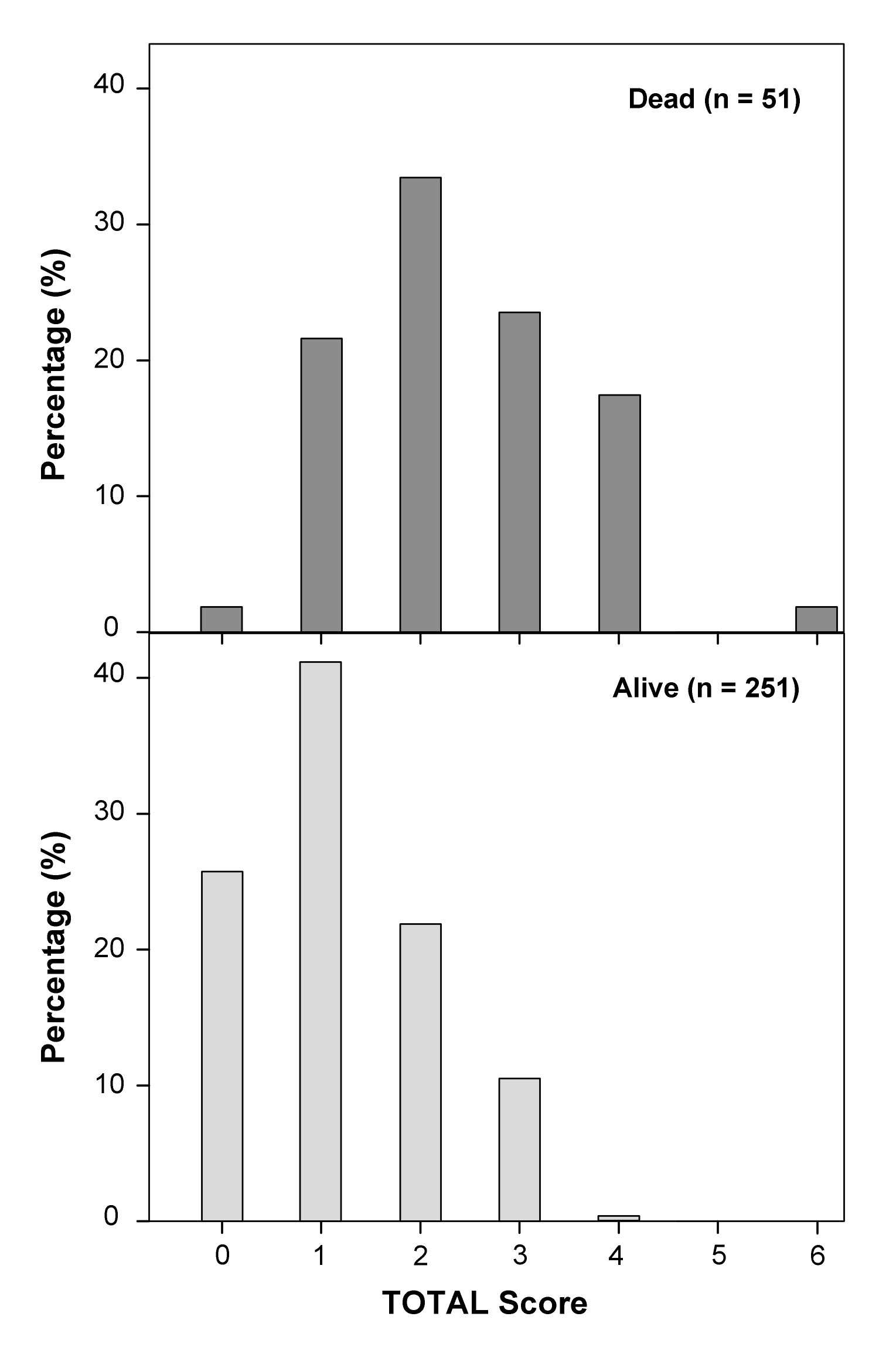

Supplement: Figure S4 — Distribution of the TOTAL scores across the two outcome groups (dead and alive); n = 302. (TIF) [file pone.0059830.s004.tif]
