# Supplementary material for: Early Warning Scores Generated in Developed Healthcare Settings Are Not Sufficient at Predicting Early Mortality in Blantyre, Malawi: A Prospective Cohort Study
Source: PLoS One. 2013 Mar 29;8(3):e59830. doi: 10.1371/journal.pone.0059830 (PMC3612104; doi:10.1371/journal.pone.0059830)
Supplement: Appendix S1 — Classification table of ECG abnormalities. (DOCX) [file pone.0059830.s005.docx]

Appendix: Classification table of ECG abnormalities:

| **Major Abnormality** |
| --- |
| Left ventricular hypertrophy |
| Left ventricular hypertrophy and strain |
| Right bundle branch block |
| Left bundle branch block |
| Left axis deviation |
| Right axis deviation |
| Q wave |
| Atrial fibrillation |
| ST depression |
| ST elevation |
| T wave inversion |
